# Prolonged Grief Disorder: A Problem for the Past, the Present, and the Future

**DOI:** 10.1371/journal.pmed.1000122

**Published:** 2009-08-04

**Authors:** Stephen Workman

**Affiliations:** Queen Elizabeth II Health Sciences Centre, Halifax, Nova Scotia, Canada

**Linked Research Article**

This Perspective discusses the following new study published in *PLoS Medicine*:

Prigerson HG, Horowitz MJ, Jacobs SC, Parkes CM, Aslan M, et al. (2009) Prolonged grief disorder: Psychometric validation of criteria proposed for *DSM-V* and *ICD-11*. PLoS Med 6(8): e1000121. doi:10.1371/journal.pmed.1000121

Holly Prigerson and colleagues tested the psychometric validity of criteria for prolonged grief disorder (PGD) to enhance the detection and care of bereaved individuals at heightened risk of persistent distress and dysfunction.

## The Past

***Mrs. Hugh Ross***

***September 1, 1918***

*…No letters from my wee soldier laddie for the last two weeks. Come on you rascal…*

***Mrs. Hugh Ross***

***October 23, 1918***

*My Friend Conners is the way my Dear Son Donald Ross spoke of you in the last letter he ever will write to me, dated “somewhere in France Aug 30th.” He was killed Sept 2nd, the day you were wounded. Donald mentioned your name in his last letter, but if I read it now I shall not be able to finish this letter to you. I will now cry out a mother's sore heart to you for some little news of my son's death and something about his last days or hours on earth. I have nothing except the cable and a letter from a Chaplain who put another man's name and number in the letter he wrote about my boy's death so it means nothing to me.*

*Perhaps you can have his little personal effects sent to me. How precious they would be to me, anything touched by his dear hands. Ah Connors may you never have that longing to see anyone that I have to see my son for I am so lonely for him and have been waiting for so long and now ah now I must wait all in vain and I loved him so.*

*Please do all you can in this for me and I shall give you a Mother's blessing.*

*Mabel Ross*

For two and a half years Mabel Ross knew nothing of her “dear son” beyond weekly letters home. Finally he wrote “…we were asked to turn in our rifles today, as we shall not require them again.” Three weeks later, seven weeks before the war's end, the cable announcing his death arrived. Donald's sister, my Grandmother, told of the impact of this news. “For years after, late at night, the house was filled with the sound of my mother's crying. It was as if she had no other children.”

Mabel Ross, still yearning to see her son, died of cancer in 1928 at the age of 55. In the parlance of the times, she died with, or perhaps of [1], a broken heart.

## The Present

In systematically, and to me at least, persuasively establishing prolonged grief disorder (PGD) as a uniquely identifiable illness that requires specific treatments, Holly Prigerson and colleagues have separated PGD from normal grief and from other forms of pathologic grief responses [2]. Is it useful to include it in the *DSM* (*Diagnostic and Statistical Manual of Mental Disorders*)? I believe it is for several reasons. First, treatment for PGD is effective and substantially different from treatment for other forms of psychiatric illnesses that can be triggered by or arise with grief and loss.

Second, and equally importantly, categorization of PGD has particular clinical relevance now. Currently, the reality that “young people may die; old people have to” is sometimes lost on families and physicians alike [3]. In the past, death occurred shortly after the development of refractory hypoxia and/or a significant decrease in level of consciousness. Now death is often a technologically supported and often prolonged experience [4],[5]. Difficulties in accurate prognostication [6] contribute to this process of care, but even in diseases where the prognosis is clear, collusion can leave many family members poorly prepared [7]. There is evidence that the aggressive treatments before death may be inversely associated with the quality of the death [8].

One particularly concerning possibility is that the current emphasis on hope and survival and “fighting” even in the final stages of disease, without also facilitating acceptance, may be contributing to the development of pathological grief reactions including PGD. I remember a middle-aged man whose father was dying; each day shorter of breath and one day closer to death. His son was continually requesting treatments his father did not want, or need. “How long do you hope for your father to live?” I asked. “I don't *ever* want my father to die.” “You can see how I am not going to meet your needs,” I replied, unnecessarily.

How this man and others like him cope after death is unknown. The opportunity for family members of patients who have died to see their family physician or some other qualified individual after six months, in order to identify and treat those suffering from PGD who wish it treated, is very appealing and somewhat comforting, at least to me.

## The Future

Improving treatment efficacy and decreasing the resources required would be especially important if estimates about the frequency of PGD are accurate [9]. If PGD lasts for many years, a backlog of individuals who could benefit from treatment exists. The establishment of diagnostic questionnaires [10] and Internet-based treatment [11] would both improve access to care. Existing hospital-based bereavement services could also assess and potentially treat PGD.

Establishing PGD as a diagnostic entity could broaden understanding about end-of-life care. Significant geographic variations in treatment intensity and integration of palliative care [12] provide an opportunity to examine the impact of illness trajectories and treatment options on the development of PGD.

Existentially, those who advocate for PGD will also always be open to criticism that they are medicalizing the unique experience of grief, but critics must concede that most deaths have become a highly medicalized experience. Establishing PGD as a DSM diagnosis will however give rise to future diagnostic and therapeutic controversies. Recent focus upon controversies [13] around the decades-old diagnosis of post-traumatic stress disorder demonstrate challenges that have arisen within a related area. Neuroimaging studies [14], not available when post-traumatic stress disorder was conceptualized, could limit controversies, enhance therapeutic understanding, and further refine diagnostic criteria. Better understanding of individuals [15] and groups [9] at risk, and of pre-death contributors to the development of PGD and other grief-related disorders [16], could enhance the opportunity for the needs of survivors to factor more fully into end-of-life care.

If in caring for those individuals dying an expected death (as most will) health care workers are not meeting the needs of those who live on, end-of-life care is falling well short of an ideal. It is in service of this ideal that Dr. Prigerson and her colleagues have characterized PGD, a historically recognized and fundamental dimension of human existence, one that I think no one would or should wish upon another. From a clinician's perspective their work in this area is rigorous, compassionate, and humane. They are to be commended for their efforts in helping to establish PGD as a unique and treatable diagnosis.

## Abbreviations

*DSM*: *Diagnostic and Statistical Manual of Mental Disorders*
PGD: prolonged grief disorder
